# Introducing our series: research synthesis and meta-research in biology

**DOI:** 10.1186/s12915-020-0755-0

**Published:** 2020-03-05

**Authors:** Shinichi Nakagawa, Julia Koricheva, Malcolm Macleod, Wolfgang Viechtbauer

**Affiliations:** 1 Evolution & Ecology Research Centre and School of Biological, Earth and Environmental Sciences, University of New South Wales, Sydney, NSW 2052 Australia; 2 Department of Biological Sciences, Royal Holloway University of London, Egham, Surrey, TW20 0EX UK; 3 Centre for Clinical Brain Sciences, University of Edinburgh, Edinburgh, EH16 4SB UK; 4 Department of Psychiatry and Neuropsychology, School for Mental Health and Neuroscience, Faculty of Health, Medicine, and Life Sciences, Maastricht University, 6200 MD Maastricht, The Netherlands

**Keywords:** Evidence synthesis, Influence synthesis, Literature synthesis, Meta-synthesis, Systematic reviews

## Abstract

Research synthesis is the process of bringing together findings and attributes from different publications, for example, to give a more complete description of phenomena than is usually possible in a single work. We bring the Research Synthesis Series to *BMC Biology* to promote meta-analyses, other research syntheses including meta-research studies, and research synthesis methodologies in biology, facilitating their dissemination to broader communities.

## Weaving biological research together

In this new Series, we provide a platform for publishing insights that arise from synthesizing existing research on biological topics, along with papers describing relevant methods for such synthesis and meta-research studies that elucidate biases, gaps and opportunities in the biological literature. While this is not the first contribution of *BMC Biology* to the topic of research synthesis [1], we strongly feel it is timely and important to establish a dedicated Series focusing on research synthesis and related methods and topics. This is because, in biology, research synthesis such as meta-analysis is, so far, primarily embraced by disciplines such as ecology, evolution and biomedical sciences, but it will certainly benefit and interest researchers in other communities in life sciences. Thus, we are truly excited to open this Series to our authors and readers.

**Fig. 1:**
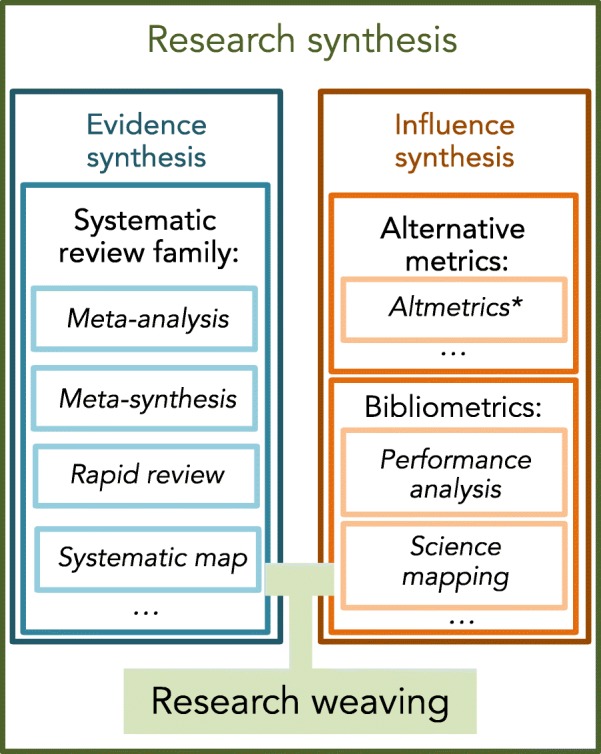
Types of research synthesis and how other types of syntheses are nested within research synthesis (see the main text for more explanation). *Altmetrics is not the type of synthesis, but Altemetrics characterize social influence or impact of a publication by counting appearances in Twitter, Wikipedia, Facebook and also policy documents (https://www.altmetric.com)

For a biologist, although it depends on the discipline, meta-analysis is probably the most familiar type of quantitative research synthesis [1, 2]. Some researchers, especially medical people, prefer using the terms ‘systematic review and meta-analysis’ or ‘systematic review and quantitative synthesis’ when they refer to a publication or study which includes a meta-analysis, which they consider to be simply the statistical analysis of material collected in a systematic review. Although these terms may be more descriptive, we feel they are a bit of a mouthful. Therefore, we use ‘meta-analysis’ as a shorthand for ‘systematic review with quantitative synthesis’ in Fig. 1, while we refer to ‘systematic review with qualitative synthesis’ (i.e., a review with qualitative interpretations offered) as ‘meta-synthesis’ [3]. Recent years have seen a marked increase in the range of approaches to systematic reviews [4], all based on a transparent, repeatable and rigorous procedure of literature search, screening and inclusion (at least in theory). This family of systematic reviews, for example, includes ‘rapid reviews’, a less comprehensive but quicker version of systematic reviews, and ‘systematic maps’ (also known as ‘evidence maps’), which catalogue related studies according to their characteristics (Fig. 1).

The members of the systematic review family are often collectively termed ‘evidence synthesis’ because they summarize the contents of related studies providing evidence for some phenomenon of interest. Another, very different, type of synthesis analyses, for instance, bibliometric information and other alternative metrics. We refer to this type as ‘influence synthesis’ to distinguish it from evidence synthesis [5]. To obtain a deeper and nuanced view of the relevant literature, it has been recently proposed to combine the use of the systematic review family with bibliometrics, such as performance analysis (e.g., total citations, *h*-index) and science mapping (e.g., citation networks, co-author networks). This novel methodological framework has been called ‘research weaving’ [5] because it weaves different components of research synthesis to give a richer fabric (Fig. 1). This new thematic section in *BMC Biology* will welcome not only any types of research syntheses, but also any associated methodologies. However, we especially welcome a synthesis or method of inter-disciplinary nature with broad significance.

## Methods and best practices in research synthesis in biology

Not only is there a dramatic increase in various types of applications of research synthesis methods, the methods and practices themselves are undergoing rapid developments. Among many such methods, statistical and computational methods associated with meta-analysis comprise a field of their own. Biologists, especially ecologists and evolutionary biologists, have made significant contributions to the meta-analytic arsenal by combining phylogenetic comparative methods and meta-analysis—‘phylogenetic meta-analysis’ where publications from different species can be aggregated in a statistically appropriate manner [1, 2]. Further, meta-analytic methods relating to genome-wide association studies (GWAS) and functional magnetic resonance imaging (fMRI) are both very distinct from standard meta-analytic methodologies which conventionally use effect size statistics such as the standardized mean difference (also known as Hedges’ *d*), the natural logarithm of odds ratio, and Fisher’s *r*-to-*z*-transformed correlation coefficient as outcome measures [6]. The methods of synthesizing GWAS and fMRI are unique contributions of biologists (geneticists and neuroscientists), statisticians and computer scientists working together. This interdisciplinarity gives much potential to research synthesis methods in biology. Importantly, there is much more work to be done for research synthesis methods to be tailored to biologists’ needs. For example, we are yet to have a meta-analytic method for integrating social network analyses, which have gained much popularity in both psychology and biology. Moreover, there is a great need to make some of the more sophisticated methods more accessible to research synthesists (e.g., via tutorials or software [7]).

We also welcome best practice and reporting guidelines for various types of research synthesis in the context of biology. Considering the significant influences of meta-analyses and other syntheses in science and beyond, it seems essential for researchers to make the quality of research syntheses consistent and high by having such guidelines. A case in point is the PRISMA (Preferred Reporting Items for Systematic Reviews and Meta-Analyses) statement. PRISMA originally aimed to improve reporting practices in meta-analyses in medical research, but is now widely used across disciplines [8]. The tens of thousands of citations they accrued over the past decade reflect their monumental impact in the community.

## Meta-research in biology

Meta-research (also known as meta-science) is the study of the process of conducting research itself, using appropriate research methodology [9]. The mission of meta-research is to provide evidence to improve scientific processes (methods, reporting, reproducibility, evaluation and incentives) by identifying and acting on research ineffectiveness, errors, biases and gaps [9]. As mentioned above, a convenient unit of research is the publication, so meta-research would often, if not almost always, use research synthesis methods, especially to obtain a bird’s eye view of scientific fields. For example, a recent work has elucidated gender imbalance and gaps in scientific authorship using > 10 million publications [10]. Notably, both methods for evidence and influence syntheses can provide such a bird’s eye view [5]. Therefore, we will also consider any contributions from meta-research in biological sciences, using appropriate research synthesis methods.

Finally, it is an absolutely thrilling time to be a research synthesist with so many research articles published every day. With your ingenuity, combined with research synthesis methods, you can synthesize and characterize biological research regardless of the topic, from microbiota to ecosystems, or from inequality to collaboration, to bring us an even better understanding of the biological world and also the world of biologists. We hope that this thematic section will be home for many research syntheses, synthesis methods and guidelines, and also meta-research studies.

## Data Availability

Not applicable.
